# Nanoanalytical electron microscopy of events predisposing to mineralisation of turkey tendon

**DOI:** 10.1038/s41598-018-20072-2

**Published:** 2018-02-14

**Authors:** Michał M. Kłosowski, Raffaella Carzaniga, Sandra J. Shefelbine, Alexandra E. Porter, David W. McComb

**Affiliations:** 1 Department of Materials and Engineering, Imperial College London, London, UK; 2 The Francis Crick Institute, London, UK; 3 Department of Mechanical and Industrial Engineering, Northeastern University, Boston, USA; 4 Department of Materials Science and Engineering, The Ohio State University, Columbus, USA

## Abstract

The macro- and micro-structures of mineralised tissues hierarchy are well described and understood. However, investigation of their nanostructure is limited due to the intrinsic complexity of biological systems. Preceding transmission electron microscopy studies investigating mineralising tissues have not resolved fully the initial stages of mineral nucleation and growth within the collagen fibrils. In this study, analytical scanning transmission electron microscopy and electron energy-loss spectroscopy were employed to characterise the morphology, crystallinity and chemistry of the mineral at different stages of mineralization using a turkey tendon model. In the poorly mineralised regions, calcium ions associated with the collagen fibrils and ellipsoidal granules and larger clusters composed of amorphous calcium phosphate were detected. In the fully mineralised regions, the mineral had transformed into crystalline apatite with a plate-like morphology. A change in the nitrogen K-edge was observed and related to modifications of the functional groups associated with the mineralisation process. This transformation seen in the nitrogen K-edge might be an important step in maturation and mineralisation of collagen and lend fundamental insight into how tendon mineralises.

## Introduction

For over 50 years, turkey leg tendon has aroused interest as a biomineralisation model^1,2^. Turkey tendons start to ossify from the 11^th^ week of the turkey’s life. The ossification proceeds in the tarsometatarsal joint region at the bone-tendon interface and progresses towards proximal regions^3^, to reach full mineralisation at 22^nd^ weeks. Age- and site-specific nucleation and growth of the mineral make turkey tendon a valuable model to study stages of the biomineralisation processes in collagen type-I tissues.

Mineralisation occurring in the turkey tendon plays an important role in enhancing the physical properties of the tendon, including the Young’s modulus, tensile strength or toughness^2,4^. Although extensive mineralisation of tendon in mammals is pathological, the mineral plays a vital role in the formation of the tendon-bone interfaces. Many aspects of the mineralisation process are similar, including: assembly of the type I collagen matrix, hydroxylation of the possible nucleation sites, formation of crosslinks, nucleation and crystallisation of the mineral into platelets^5,6^. A more complete appreciation of the mineralisation process in tendon will inform our understanding of how mineral forms in collagenous tissues.

In turkey tendon, collagen fibrils grow in diameter with age^7^. As the tendon calcifies, individual fibres fuse into thick (>100 µm-wide) beams^2,8^. At the fibrillar level of tissue hierarchy, the non-mineralised, partially and fully mineralised regions are present in close vicinity to each other^9^. The mineral observed in turkey tendons, in the early mineralisation stage, has been described as amorphous calcium phosphate (ACP) by analogy to the mineral formations detected in zebra fish bone^10^ and *in vitro* mineral studies^11^. The mineral in the advanced mineralisation stage has been identified as crystalline apatite^2^.

There are various types of mineral formation observed in the mineralising turkey tendon. Large (>150 nm in diameter), extracellular vesicles containing clusters of misaligned crystallites have been observed^2,12,13^. By analogy to the *in vitro* studies^11,14^, it is suggested that vesicles containing amorphous mineral attach to the collagen fibril and form mineral clusters, which act as a mineral store^15^ and that the disorganised crystallites align within the collagen fibrils^13^. Clusters of small (10–20 nm long), scattered crystallites have also been observed on the surface of the collagen fibrils^13,16^. It is not clear if these different organisations of mineral relative to the collagen fibrils reflect different mineralisation pathways or whether they are connected. The evidence provided in the literature is also not clear about the physiochemical format of the mineral which is delivered to the collagen matrix, whether it is present as diffused ions, vesicles of amorphous calcium phosphate or clusters of disorganised apatite crystals.

The chemical composition of collagen and mineral are usually assessed by bulk methods, and the site-specific chemistry of each constituent at the nanometer scale is still poorly understood. In particular, age-dependent evolution of the chemistry of the bone-tendon and proximal-distal tendon transitional regions during tendon formation and growth require further investigation^17^. Changes in the chemistry of the collagen fibrils during the early stages of tendon mineralisation have not been related to their arrangement and structure at the nano-meter scale^18^. Some studies highlight the presence of non-collagenous proteins (i.e. phospoproteins and carboxyglutamic acid) within the mineralising tendon^19,20^. Other studies suggested that turkey tendon itself undergoes both structural and chemical changes prior to mineralisation^18,21^. These changes result in a more organised collagen matrix and increase in the amide III and CH_2_ bands examined by Raman spectroscopy^22^. However the spatial resolution of this Raman spectroscopy study is limited to few micrometres.

We conducted a combination of electron tomography and spatially resolved electron energy-loss spectroscopy (EELS) of cryogenically fixed mineralised fibrils in order to determine how the chemistry and morphology of the dominant mineral-collagen assemblies changed during formation and growth. To study this process, tendon tissues from 11-, 14- and 22-week old of turkeys to identify the relevant spectral and morphological features associated with mineralisation in different ages.

## Results

### Structural and chemical changes in mineralising turkey tendon

Examination of the turkey tendon revealed that tissues at different stages of mineralisation may be found within the same section, regardless of the age of given specimen. Figure SI1 shows bright-field TEM images of representative regions of non-mineralised and mineralised turkey tendon collagen of all three age groups.

**Figure 1:**
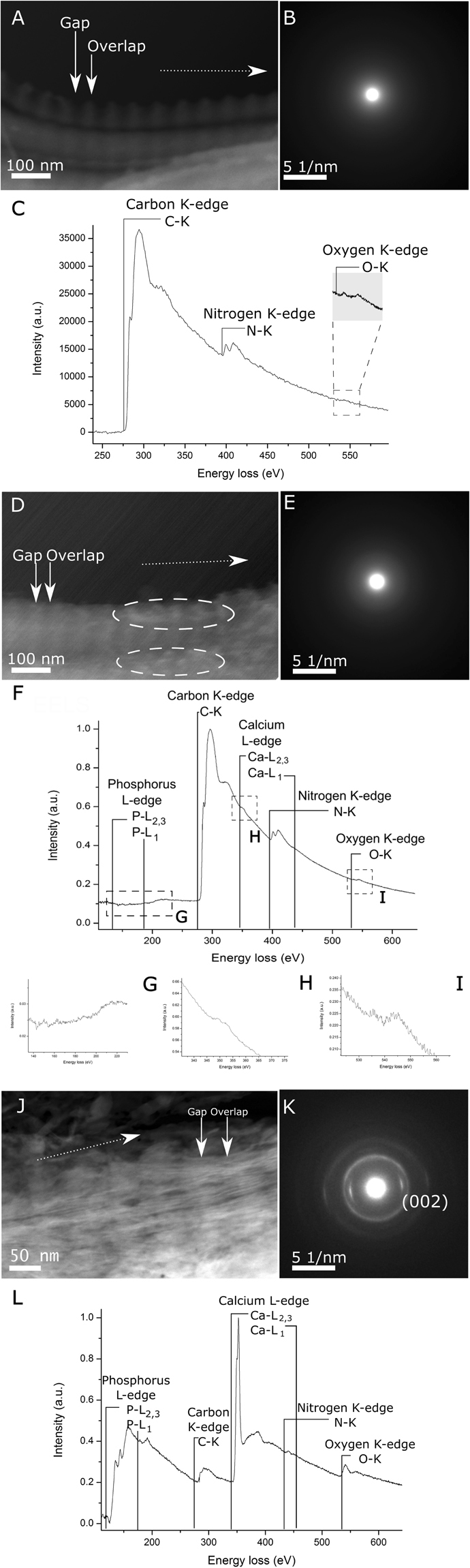
Characteristic features of non-mineralised (**A**,**B**,**C**), poorly mineralised (**D**,**E**,**F**,**G**,**H**,**I**) and well mineralised (**J**,**K**,**L**) regions of turkey tendon. (**A**) Non-mineralised region shows banded fibrils with mineral absent. Dotted arrow indicates the direction of the periodic banding pattern. (**B**) SAED shows a diffused halo characteristic of amorphous material. (**C**) An EELS spectrum of a non-mineralised turkey tendon collagen fibril showing characteristic carbon (starting at 285 eV), nitrogen (at 400 eV) and oxygen (at 530 eV) K-edges. (**D**) Poorly mineralised region exhibits banded fibrils with ellipsoidal granules (dashed regions). In regions with these granules, the banding contrast is difficult to resolve. (**E**) SAED taken from the fibril shows a diffused halo attributed to amorphous material. (**F**) EELS spectrum shows the presence of carbon (starting at 285 eV), calcium (H, at 345 eV) nitrogen (at 400 eV) and oxygen (I, at 530 eV) edges. The phosphorus signal (**G**) could not be properly examined due to the poor signal-to-noise, but irregular features in the spectrum between 130–160 eV suggest presence of phosphorus. Close-ups of calcium and oxygen signals could be seen on respective insets (**H**) and (**I**). (**J**) The well mineralised region contained banded fibrils and mineral crystals. (**K**) SAED acquisition shows a ring pattern typical for apatite. The (002) plane reflections are indexed. (**L**) EELS spectrum shows the presence of phosphorus (starting at 125 eV), carbon (at 285 eV), calcium (at 345 eV), nitrogen (at 400 eV) and oxygen (at 530 eV) edges.

Regardless of the age group, regions at three different stages of mineralisation were observed. In the non-mineralised regions (Fig. 1A), only collagen fibrils could be seen. No mineral granules or crystals were seen. SAED patterns did not show any crystal plane reflections (Fig. 1B) and no elements characteristic of calcium phosphate mineral were visible in the EELS spectra. Only signals characteristic of collagen fibrils (simultaneous presence of carbon, nitrogen and oxygen) were recorded (Fig. 1C).

In poorly mineralised regions, ellipsoidal granules were found (Fig. 1D), which resemble the mineral nucleation clusters described in *in situ*^23^ and *in vivo*^13,16,24^ studies of the bone mineralisation process. The granules were usually aligned in the gap regions of collagen fibrils, forming larger clusters, or along the surface of the fibril. These formations were very sensitive to beam damage and could be easily destroyed during the imaging process. SAED patterns taken from the poorly mineralised regions did not show any presence of a crystalline structures (Fig. 1E). However, EELS detected mineral elements (phosphorus, calcium, oxygen) in the regions containing amorphous granules at very low intensity (Fig. 1F,G,H,I).

In well mineralised regions of tendon, crystals were clearly visible (Fig. 1J) and their crystallinity was confirmed by SAED patterns (Fig. 1K). These regions displayed strong EELS peaks for mineral elements (phosphorus, calcium). All well mineralised parts of the tendon showed the presence of phosphorus, carbon, calcium, nitrogen and oxygen (Fig. 1L).

### Evolution of the mineral phase

**Figure 2:**
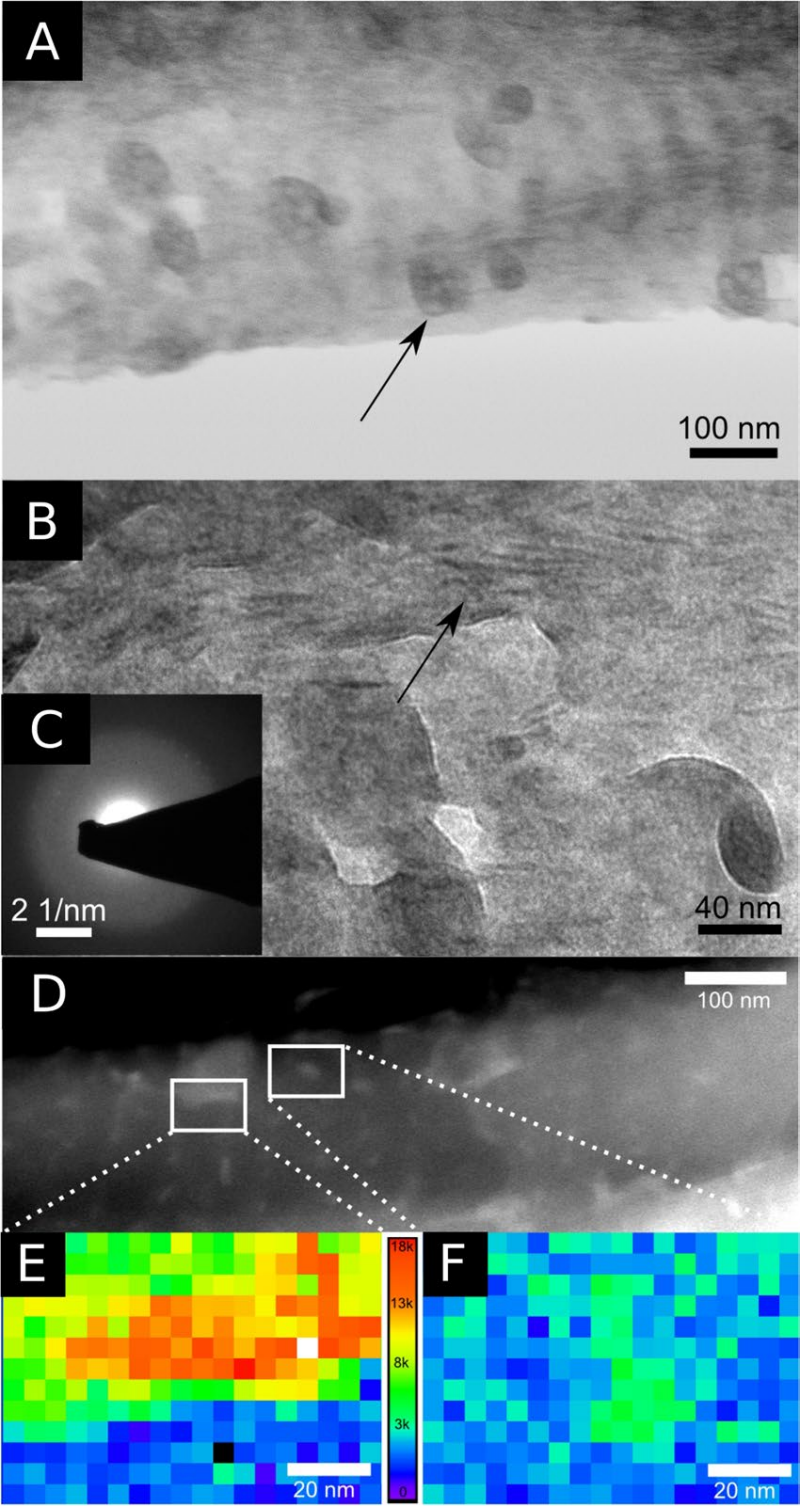
TEM images of a poorly mineralised region of 14 week old turkey tendon showing (**A**) large clusters and (**B**) needle-like granules. The insert shows the corresponding (**C**) SAED pattern characteristic of an amorphous material. (**D**) ADF-STEM image of a poorly mineralised fibril showing calcium-containing clusters and granules. A false-colour calcium intensity map (a.u.) of (**E**) a large cluster and (**F**) a single granule. Regions with higher intensities of the calcium signal are “hotter” (red and orange), while regions with low intensities are “colder” (blue and violet).

In the poorly mineralised regions, large clusters of amorphous mineral (diameter 46.1 ± 13.8 nm, n = 47, Fig. 2A) and small elongated granules (Fig. 2B) were observed. Although SAED diffraction patterns taken from regions similar to seen on Fig. 2A,B are consistent with an amorphous material (Fig. 2C), EELS showed that there is an increase in the calcium signal from both, the large clusters and the ellipsoidal granules. In Fig. 2D,E,F, intensity maps of calcium present in regions with clusters and granules are shown. In Figure SI2, summed non-processed spectra from these regions of interest (clusters, granules and their surrounding) are presented showing the presence or absence of Ca-L_2,3_ edge features (white lines). While hints of phosphorus presence could be seen in these regions (Fig. 1F,G), the signal was too weak to obtain reliable intensity maps.

**Figure 3:**
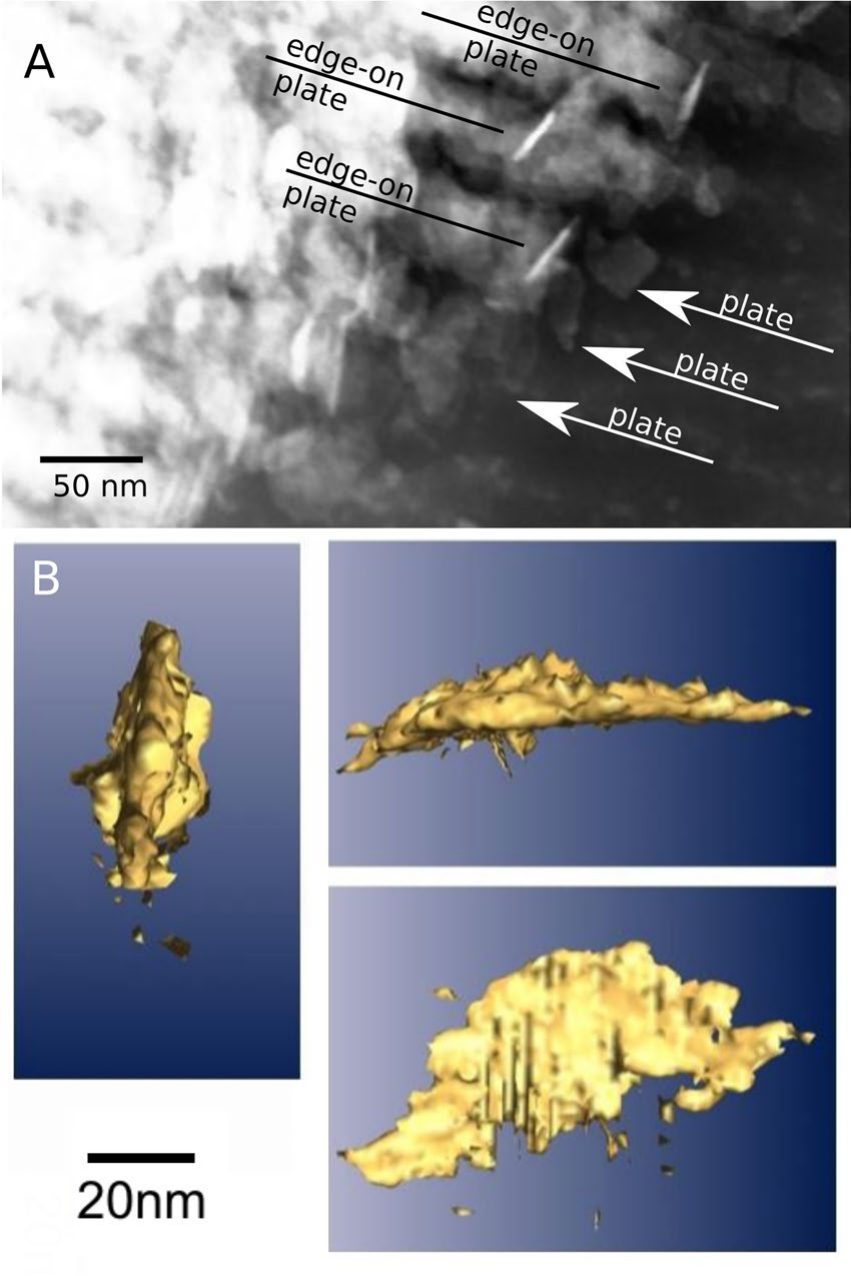
(**A**) The well mineralised tissue cut at an oblique angle to the fibril plane revealed plate-like mineral crystals and needle-like impressions which are platelets oriented edge-on. (**B**) A volume rendering of a tomographic reconstruction of a mineral plate within the gap region of turkey tendon.

In the well mineralised regions, crystals were found and their crystallinity was confirmed by SAED patterns (Fig. 1K). The crystals had a similar size and shape to those observed in bone (Figure SI2)^25^. Mineral crystals were visible on 2D micrographs either as sharply contrasting needle-like objects or less contrasting platelets (Fig. 3A). Electron tomography (Fig. 3B) confirmed that all crystals in 3 examined regions develop into a plate-like form and the needle-like shape of crystals seen in 2D images originates from platelets observed edge-on.

No changes were observed in the EELS fine structure of phosphorus and calcium L-edges between the poorly mineralised and well mineralised tissues (Figures SI3 and SI4). As there were no changes in the observed EELS spectra related to structural features (*e.g*. collagen banding, presence/absence of clusters), the best representative spectra were selected from the various areas. Differences were detected in EELS fine structure from these regions at the carbon K-edge, nitrogen K-edge and oxygen K-edges, which are described in the subsequent section.

### EELS of the carbon K-edge

**Figure 4:**
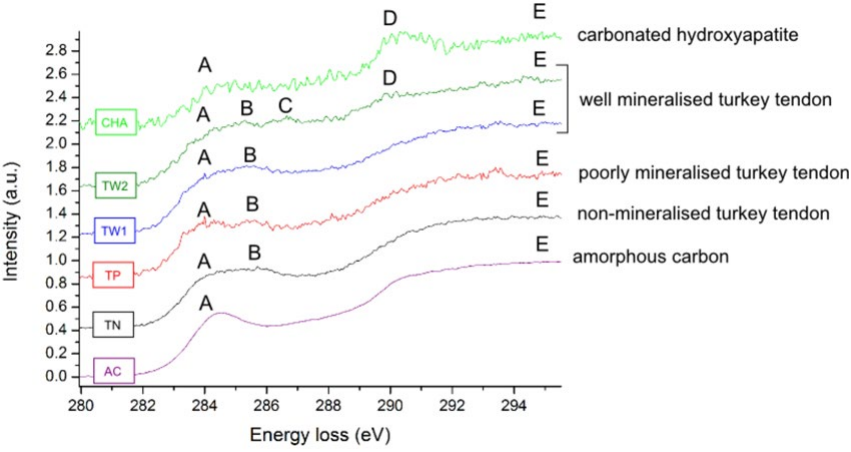
The carbon K near edge structures of turkey tendon fibrils (collected from 14 week old specimen), carbonated HA (CHA) and amorphous carbon (AC). The amorphous carbon (AC) spectrum is consistent with spectra collected from resin and carbon film. The AC spectrum has two peaks: a smaller peak A at ~284 eV and a broad peak E above 292 eV. Spectra collected from turkey tendon (TN, TP, TW1, TW2) display an additional peak B at ~286 eV. The mineralised regions show also a formation of peak C at ~287 eV (TW2). In some acquisitions, (TW2), a carbonate peak D at ~290 eV is seen.

Carbon K-edges of non-mineralised and mineralised collagen fibrils (Fig. 4) were compared with carbon K-edges of the amorphous carbon film support (AC) and carbonated hydroxyapatite (CHA)^26^.

Carbon spectra were normalised and aligned to the first peak A, which was set to an energy-loss of 285 eV, based on the literature^27^. Spectra of non-mineralised (TN) and poorly mineralised (TP) regions exhibit three characteristic peaks A at ~285 eV, B at ~286 eV and a broad structure E above ~292 eV. Some spectra of well mineralised regions (TW2) show additional peaks C and D at ~287 and ~290 eV, respectively.

Peak A is assigned to 1s-π* transitions in sp2-like carbon species^28^. Assignment of peak B is less definitive. X-ray absorption spectroscopy (XAS) has shown that a peak in this region can arise from carbon or carbonyl groups in an aromatic conformation^29–32^. This could be due to aromatic rings, possibly in collagen-forming nucleic acids, like phenylalanine and tyrosine. Other XAS studies attributed peak B to 1s-π* transitions in a nitrated carbon structures^33^. This could be due to collagen-forming amino acids, but could also be associated with collagen crosslinking (pyridine, pyrole). In the present study, the peak at ~288 eV associated with 1s-π* transitions associated with –CN groups was not observed^33^.

There is a variation in spectra collected from the well mineralised regions. In some regions (represented by TW2), peak C and D were observed. Theses peaks are difficult to resolve and interpret. Peak C can be attributed to 1s-σ* transitions in an aliphatic^34^ or a diamond-like bond^35^, or 1s-π* transitions in peptide bonds in carbonyl^30,35^ or amidyl^34^ and 1s-π* transitions in carboxyl^34^. As features at this position could not be fully assigned, any association with mineral-collagen bonding, amino acids or crosslinking is highly speculative. Spectrum TW2 shows also a fine structure D, which is characteristic of 1s-π* transitions in carbonate groups^36,37^. This feature is clearly visible in the carbonated HA standard (CHA). All spectra exhibit a broad peak E assigned to 1s-σ* transitions from C-C bonding^28^.

In summary, all carbon edges collected from turkey tendon exhibit peaks A and B (Fig. 4) characterised previously as arising from the collagen fibril. The carbon K-edge spectra from the poorly mineralised region (TP) exhibited a strong resemblance to spectra collected from non-mineralised fibrils (TN) and with some regions where mineral crystals were observed (TW1). However, the carbon K-edge of the well mineralised fibril (TW2) typically shows peaks characteristic for mineral, *i.e*. a carbonate peak at ~290 eV, and additional peak of unclear origin at ~287 eV.

### EELS of the nitrogen K-edge

**Figure 5:**
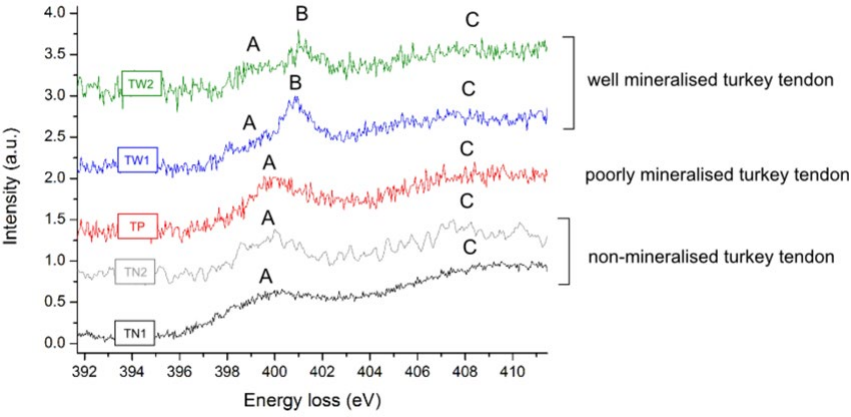
The nitrogen K near edge structures of non-mineralised (TN) and mineralised (TP, TW) 14 week old turkey tendon fibrils. In the nitrogen spectra, three peaks can be observed: A at ~400 eV, B at ~401 eV and a broad peak C at ~408 eV.

The nitrogen signal is used to confirm the presence of protein, and as such, this element was not observed in any of the mineral standards. In the nitrogen K-edge, three peaks were observed: A at ~400 eV, B at ~401 eV and C at ~408 eV (Fig. 5). Nitrogen spectra were aligned using calcium L_2,3_-edge calibration and normalised to the most intense peak of the nitrogen edge. The calibration of energy scale of non-mineralised regions was confirmed by observation of the zero loss peak before and after the acquisition.

Non-mineralised and poorly mineralised fibril spectra (TN1, TN2, TP) displayed two peaks A and C (Fig. 5). In well mineralised tissues (TW1, TW2), the nitrogen K-edge shows an additional peak B. There is a significant difference in the intensity of peaks A and B.

Peak A may be attributed to 1s-π* transitions characteristic for nitrogen in an aromatic ring, especially pyridine^32,38–40^, which is essential part of collagen crosslinking, or more generally with transitions in nitrated carbon groups^33^. Peak B might be connected with oxidised pyridine^32,38^, 1s-π* transitions in the nitrated carbon structure^33,39^ or 1s-π* transitions in glycine amide groups (C = ONH)^41^. The broad peak C is attributed more generally to 1s-σ* transitions in amino compounds^32^.

Spectra of the nitrogen edge collected from non-mineralised (TN1, TN2) and poorly mineralised (TP) fibrils are comparable. Spectra collected from the well mineralised tissues (TW1, TW2) display a clear separation in two peaks: A at ~400 eV and B at ~401 eV. This split may show energy shifts between 1s-π* transitions in pyridine and oxidised pyridine, respectively^38^. A broad peak C corresponding to 1s-σ* transitions in the amino groups is also observed.

### EELS of the oxygen K-edge

**Figure 6:**
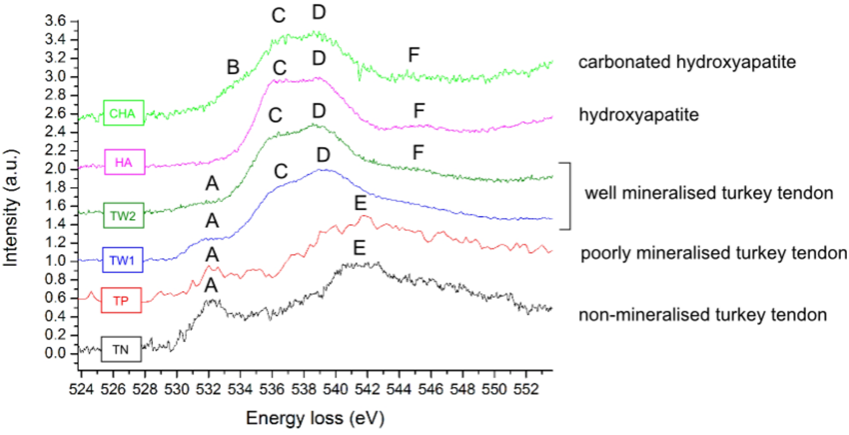
The oxygen K near edge of non-mineralised (TN) and mineralised (TP, TW) 14 week old turkey tendon fibrils, and hydroxyapatite (HA) and carbonated HA (CHA) standards. In mineralised tissues, a mixture of features characteristic of the mineral and collagen can be seen.

The oxygen K-edge of non-mineralised and poorly mineralised regions displays two peaks: A at ~532 eV and E at ~543 eV (TN, TP, Fig. 6). Oxygen K-edges in the well mineralised (TW) regions show strong, apatite-like features: a double peak C-D at ~537 and ~539 eV, respectively and a weak peak F at ~545 eV. Mineralised tissues also display smaller features A and E, which are fully resolved in spectra of non- and poorly mineralised regions.

Oxygen spectra of mineralised regions were aligned using peak D, which was set to an energy-loss of 539 eV^26^. The calibration of energy scale of non-mineralised regions was confirmed by observation of the zero loss peak before and after the acquisition. Spectra were normalised to the most intense peak of each individual spectrum.

Peak A is attributed to 1s-π* transitions in carboxyl or amide groups^32,34^. The shoulder B in carbonated HA was attributed to 1s-π* transitions in carbonate groups and peak C to 1s-π* transitions to final states associated with Ca-O hybridisation^42^; however, in mineralised tissues, there is an overlap of signal coming from protein. Peaks characteristic of organic polymers should form in 530–536 eV region^32^, which correspond to position of peaks B and C. In organic material, features at the B position originate from 1s- σ* transitions in C = ONH or COOH groups^32,41^, and features at the C position originate from 1s-π* O-H transitions in COOH^41^ or 1s- σ* transitions in C-O-C or O-C-N groups^32^.

The assignment of peak D possibly it originates from transitions to calcium-oxygen or phosphorus-oxygen orbitals in mineral^42^. In organic material, the broad structure E is attributed to 1s- σ* transitions in O-H groups, while the higher energy shoulder F comes from 1s- σ* transitions in C-O groups^32,41^. In mineral, the assignment of peak F is also not clear. In oxides, peaks of similar energy-loss are attributed to transitions to states associated with calcium-oxygen bonds^42^.

Spectra recorded in non-mineralised (TN) and poorly mineralised (TP) regions could be attributed to amino acids, most probably glycine^41^, which is the most common acid in the collagen chain. In well mineralised tissue (TW), spectra are dominated by strongly resolved apatite-like features (peaks C, D and F), but features characteristic of organic polymers may also be seen. Formation of peak A could be attributed to the presence of proteins, especially glycine, and shoulder E is relatively more intense in tissues than in mineral standards.

## Discussion and Conclusions

Nanoanalytical electron microscopy techniques were able to differentiate between non-mineralised and mineralised tissues at three consecutive stages of development (before, during and after mineralisation) on the nanometer scale. The chemical and structural composition and structure of the turkey tendons varied between regions of different mineralisation stage (non-mineralised, poorly mineralised and well mineralised). These three types of regions were classified on the basis of their morphology assessed *via* micrographs, crystallography assessed *via* SAED and chemistry assessed *via* STEM-EELS.

There is a significant inconsistency in the nomenclature and description of the mineral formations in mineralising tissues. In the present study we use the following definitions, ‘vesicle’ refers to a phospholipid sphere containing amorphous mineral precursors, typically 150–500 nm in diameter; ‘cluster’ refers to a smaller (50–150 nm in diameter) mineral formation or aggregate; ‘granule’ refers to a small (10–20 nm) mineral formation.

In non-mineralised regions (consisting of collagen only), no morphological, crystallographic or chemical indicators of the mineral presence could be observed. In poorly mineralised regions, diffuse, calcium/phosphorus-containing granules and clusters were visible; SAED patterns did not reveal the presence of any ordered, crystalline structure at this scale. In well mineralised regions, well-defined crystals were frequently observed. The well mineralised regions were crystalline and the EELS signatures could be assigned to carbonated apatite. Characterisation of these three discrete structures provides important insight into how the mineral nucleates and grows on the collagen fibrils during tendon calcification.

No significant changes in EELS fine structures could be observed between the non-mineralised and the poorly-mineralised areas, with the only difference being some hints of the presence of Ca and P in poorly mineralised regions. By contrast, the well-mineralised tissue presents well-differentiated fine structure, which resembles that of carbonated HAP observed in similar conditions. We did not observe any variation in the fine structures that could be related to structural features such as gaps and overlaps of collagen fibrils or regions with and without the mineral precursor. The clear separation of modifications in the fine structures seen between the poorly and well mineralised regions suggests that the modification did not occur before the nucleation of mineral and are potentially triggered by the presence of mineral precursors. Most significant modifications were observed in the nitrogen and oxygen K-edges. In the nitrogen K-edge, fine structure with a peak at ~400 eV observed in non-mineralised and poorly mineralised regions, evolves into a double peak structure in well mineralised regions. Although the transformation seen in the nitrogen K-edge might be an important step in the mineralisation of collagen, the origin this transformation is not unequivocal. In the oxygen K-edge, fine structures in non-mineralised and poorly mineralised regions are comparable to spectra recorded for proteins^41^. In well mineralised regions, fine structure of protein is overlapped by signal coming from mineral. Although the changes in the O K-edge of fully mineralised turkey tendon appear to be consistent with spectra collected from mature bone^43^, in turkey tendon the organic component contributes more to the final spectra. Therefore the differences in the O K-edges come mainly from the protein/mineral ratio and the composition of mineral (especially the presence of carbonate content).

In the carbon K-edges of all regions, peak at ~286 eV, characteristic to aromatic carbon structures, was seen. Occasionally, in well mineralised region two new peaks at ~287 and ~290 eV appeared. Three peaks were assigned to transitions in carbonyl/carboxyl groups and to transitions in carbonate, respectively. The presence of those peaks might be related to changes in collagen during mineralisation and to nucleation of the mineral.

Although the phosphorus and calcium L_2,3_-edges did not show significant variation in their shape between different regions, a variation in the intensities of these edges was observed. In the non-mineralised regions, the presence of phosphorus and calcium was not detected. In poorly mineralised regions, weak signals characteristic of phosphorus and calcium were recorded and in well mineralised regions, very intense signals were detected. As there is mixing of collagen and mineral phases in the developing tissue, a more holistic approach (observation of few edges at the same time) might be beneficial to the identification of signal origins and assignation of spectral features to specific chemical structures.

Assessment of turkey tendon chemistry by EELS revealed signatures, which might be characteristic of the presence of nucleic acids with aromatic structures in collagen, mature collagen crosslinking and/or other associated proteins. The core loss edges showed features possibly originating from pyridine-based amino acids. With application of monochromated EELS, there is a possibility of further unravelling of the collagen chemical signature and mapping the distribution of aromatic structures in fibrils at different development stages and in pathologic tissues.

The turkey tendon model provides insights into the structure of collagen type-I based tissues. Stages of mineral development in turkey tendon are fairly straightforward to assess in the bulk of the sample. Statistically significant variations between different age groups were measured in bulk assessments of the Ca/P ratios, the mineral crystallinity and composition, and the protein content. However, characterisation of mineral development at the nanometer scale is a challenging task, as the mineralisation front is very disperse and regions at different stages of development can be seen in each sample. Our TEM analysis shows that in each age group, the tissue contains regions at different stages of development, rather than from a homogeneous development of the whole tissue. Future work is needed to understand whether such inhomogeneous development within the tissue is generalizable to other tissues such as bone. Fish bone and fish scale models have also been examined in context of biomineralisation^10,44^. Fin bone calcifies from the roots to the tip, similarly to turkey tendon. Micrometer scale studies suggest that fish bones mineralisation more homogeneously with age than in turkey tendon and might be tracked at nano-scale level^45^.

The *in vivo* mechanisms by which mineral ions are delivered into the collagen matrix, are still under debate. It has been suggested that the mineral is delivered from cells to fibrils in amorphous calcium phosphate vesicles or clusters of disorganised apatite crystals. We observed small (~50 nm) clusters of amorphous calcium phosphate in poorly mineralised regions and clusters of disorganised apatite crystals, which have been suggested to be a transient precursors to mineralisation of the collagen fibrils^2,14^, in the well mineralised regions. Both types of clusters are structurally similar to primary and secondary fetuin-mineral complexes, known as calciprotein particles (CPPs)^46,47^, and prenucleation clusters^48^ reported in various *in vitro* studies. Although the *in situ* model suggests that the cluster formation and mineral nucleation begins, when the body fluids in the vicinity of collagen fibrils are super-saturated with calcium and/or phosphate ions^48,49^, formation of CPPs might enhance this process^50^.

To our knowledge this is the first study which supports the presence of CPPs or prenucleation clusters in mineralising tissues *in vivo*. Previous *in vivo* observations of disorganised clusters of well crystallised mineral in partially mineralised fibrils^12,13,15^ could be the artefacts of the non-anhydrous preparation method (*i.e*. mineral crystallisation). Also the presence or absence of mineralising cells in examined region of the turkey tendon may have an impact on the distribution and evolution of the mineral^14,45^.

**Figure 7:**
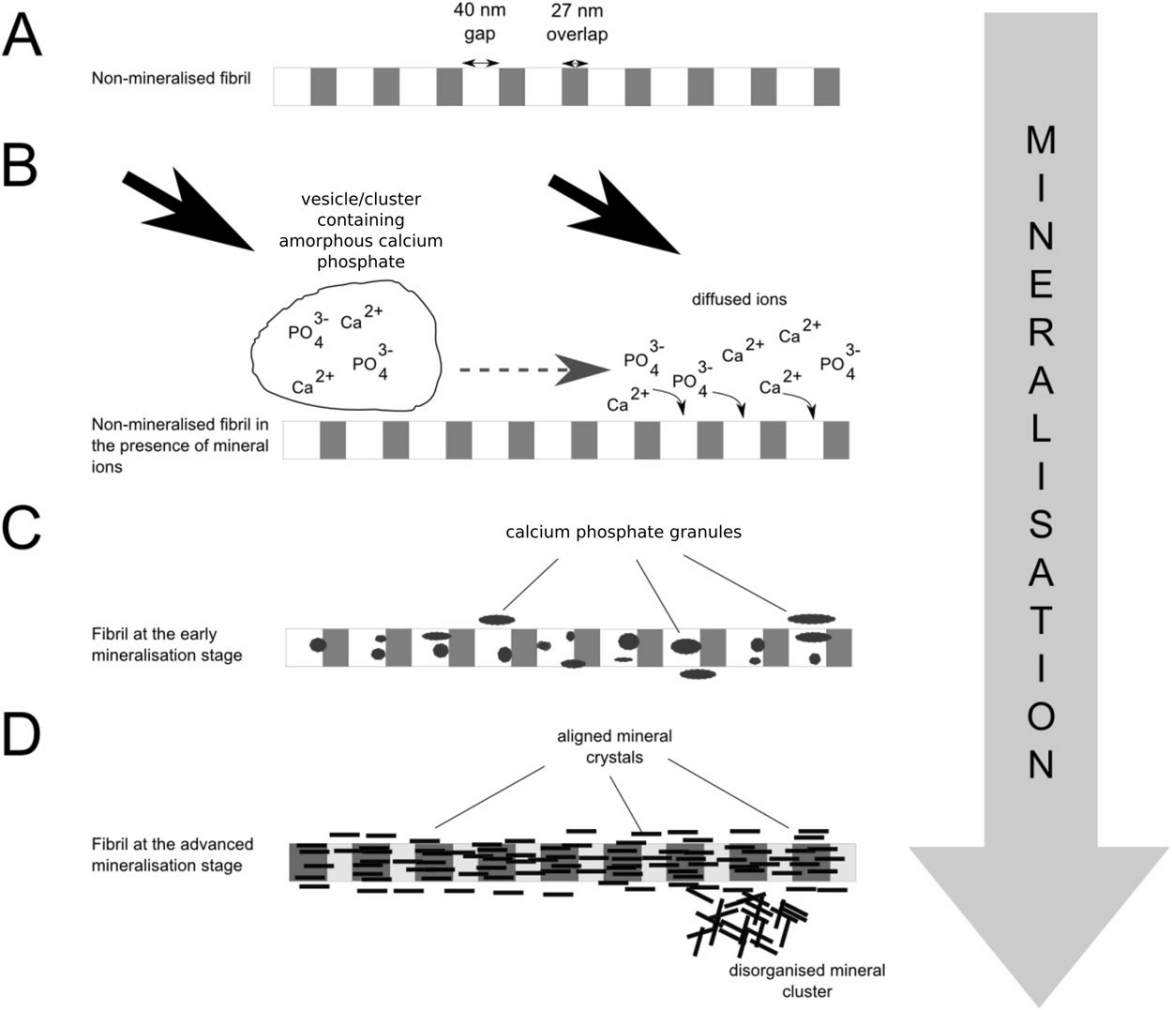
Schematic showing the evolution of mineral based on various mineralisation studies. (**A**) A non-mineralised fibril. (**B**) The mineral ions are delivered into the collagen matrix either as vesicles/clusters of amorphous calcium phosphate, which dissolve and release the ions, or free ions are transported in the body fluids. (**C**) Mineral ions start to aggregate forming mineral granules in the gap regions. The periodic banding contrast starts to reverse. (**D**) Mineral granules crystallise into aligned crystals inside, and outside, the fibril. During development some of the extrafibrillar crystals may lose their alignment and rearrange into disorganised clusters.

We hypothesise that mineral ions are delivered into the collagen matrix, in which clusters of amorphous mineral are formed (Fig. 7). The cluster formation process may start within the vesicle, occur spontaneously after dissociation of the vesicle^14^ or be governed by the non-collagenous proteins^46,50^. Mineral ions released from clusters into the collagen matrix nucleate and grow, preferentially in the gap regions of fibrils^23^. Initially, calcium/phosphorus ions form amorphous granules, which crystallise and grow with time. The crystallisation process may occur in the intra- and extrafibrillar regions^23^. The mineral nucleating inside fibrils then aligns its crystallographic c-axis parallel to the long axis of collagen^13,51^. Mineral nucleating outside fibrils usually follows the same alignment, but with limited support from fibrils or in the absence or presence of specific non-collagenous proteins extrafibrillar mineral may arrange into disorganised clusters of crystalline mineral^23,52^. Observations of Ca-containing granules and changes of EELS signatures at different stages of mineral development fit well into the current model of collagen mineralisation summarised above. In addition, our work paves the way for application of nanoanalytical spectroscopy to understand the earliest stages of mineralisation at hard-soft tissue interfaces, which could provide vital insight into how to repair these interfaces in damaged tissues.

## Materials and Methods

To characterise the collagen and mineral development, tissue samples of Achilles tendon were collected from turkeys of three age groups, which are predicted to reflect different mineralisation stages: early (11-week old), intermediate (14-week old) and advanced (22-week old)^7,18^. Fresh tissues of culled turkeys 11, 14, and 22 weeks old (Norfolk White breed, all females) were acquired from a local farmer. Turkey tendon samples for TEM were prepared by both, high pressure freezing and freeze substitution (HPF/FS) and anhydrous methods, to ensure the adequate preservation of mineral and collagen in tissue^14,53^. Embedded material was sectioned, using an ultra-microtome.

200 µm-thick samples of turkey tendon were cut, mounted in flat specimen carriers with 1-hexadecene and transferred into a Leica EMPact2 (Leica Microsystems, Vienna) machine for high pressure freezing (HPF). A Leica EM AFS2 machine cooled to −90 °C was used for 8 h-long freeze-substitution (FS). The acetone-based substitution solution contained 3% (v/v) glutaraldehyde. After 8 h, the samples were warmed to 0 °C at a constant rate of 5 °C/h. Before reaching the room temperature, samples were washed twice in 100% acetone for 15 min.

Tendon samples were dipped successively in 1:3, 1:1 and 3:1 resin:acetone solutions for 24 h each time. The prepared resin was a mixture containing of 33.6% of Quetol651, 47.7% of nonenylsuccinic anhydride (NSA), 16.6% of methylnadic anhydride (MNA) and 2.1% of benzyldimethylamine (BDMA), (Agar Scientific, Dorset, UK). For 7 days, samples were immersed in pure resin under vacuum and the resin was changed daily. On day eight, the samples were placed in the curing oven and heated at 60 °C for 48 h.

An ultramicrotome Power Tome XL with an ultra 45° Diatome diamond blade was used to prepare thin (70 nm) sections of embedded samples. An automated procedure was employed with cutting speeds of 0.5 mm/s. A specific cutting speed was selected to allow sufficient time for relaxation of a section of material taken from the block face. The knife was set to a 6° angle to the front of specimen.

Bright-field transmission electron microscopy (TEM) of turkey tendons were performed on a FEI Titan TEM operated at 300 kV and fitted with a Gatan Tridiem electron energy-loss spectrometer (EELS). The microscope was operated in scanning transmission electron microscopy (STEM) mode for the EELS measurements. A 50 µm condenser aperture, a spot size 9 (a probe size of ~1 nm) and a 48 mm camera length were used to optimise the signal-to-noise ratio. In these conditions, the EELS collection semi-angle was 14 mrad and the STEM probe convergence semi-angle was 8 mrad. The core loss signal was acquired in 10-second exposures utilising sub-pixel scanning with a total electron dose less than 10^4^ electrons/nm^2^ to prevent electron beam damage. Spectra were collected with an energy resolution of 0.6–0.7 eV, using an energy dispersion of 0.05–0.1 eV/channel, and a spatial resolution of 5 nm. After a background subtraction, spectra were aligned to the characteristic (usually first) peak and normalised to the most intense peak. Mineral standards spectra collected in a previous study^26^ were used as a reference.

For the Ca- L_2,3_-edges, an accurate background could not be subtracted due to the presence of the tail of the preceding carbon K-edge, instead a 10 eV-wide background fit was used. To obtain intensity maps, the calcium signal was integrated over 10 eV-wide windows (345–355 eV). To validate this approach, we show non-processed summed spectra from respective regions of interest in Supplementary Information.

To reconstruct the morphology of the mineral, electron tomography acquisitions of the intrafibrillar mineral were taken at 2° steps from −50° to 50° in the STEM mode using a high angle annular dark field detector (161–681 mrad). The total dose for the imaged region was estimated around 2*10^4^ electrons/nm^2^. After the experiment a loss of crystallinity was observed, but the changes in morphology were negligible. 3D reconstruction was performed, using Inspect 3D processing software, and visualised, using Amira 3D software (FEI, Netherlands). A solid isosurface was used to represent individual mineral platelets.

### Data availability statement

The datasets generated and analysed during the current study are available from the corresponding author on reasonable request.

## Electronic supplementary material

Supplementary Information
