# Supplementary material for: Nanoanalytical electron microscopy of events predisposing to mineralisation of turkey tendon
Source: Sci Rep. 2018 Feb 14;8:3024. doi: 10.1038/s41598-018-20072-2 (PMC5813010; doi:10.1038/s41598-018-20072-2)
Supplement: Supplementary file 1 — Supplementary Information [file 41598_2018_20072_MOESM1_ESM.pdf]

Nanoanalytical electron microscopy of events predisposing to mineralisation of turkey tendon

Supplementary information

Authors:

Michał M. Kłosowski[1*], Raffaella Carzaniga[2], Sandra J. Shefelbine[3], Alexandra E. Porter[1]

David W. McComb[4*]

[1]Department of Materials and Engineering, Imperial College London, London

[2]The Francis Crick Institute, London

[3]Department of Mechanical and Industrial Engineering, Northeastern University, Boston

[4]Department of Materials Science and Engineering, The Ohio State University, Columbus

*Correspondence to mccomb.29@osu.edu or mmklosow@ic.ac.uk

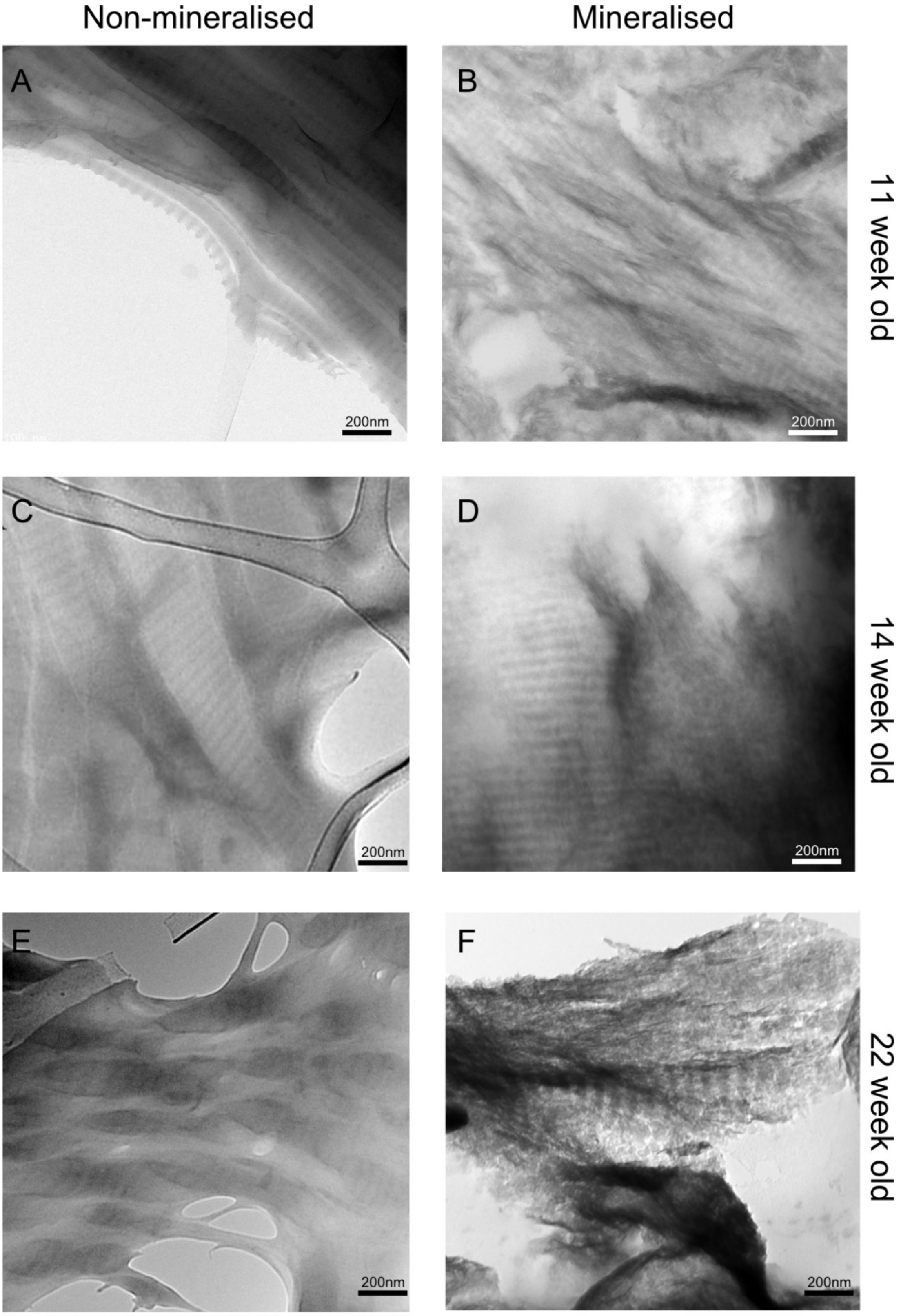


**Figure SI1 Bright field TEM images of non-mineralised (A, C, E) and mineralised (B, D, F) turkey tendon. In non-mineralised tendon, the darker contrast arises from the overlap region, which contains overlapping collagen molecules. In mineralised tendon, the darker contrast arises from the gap region, which contains mineral crystals.**

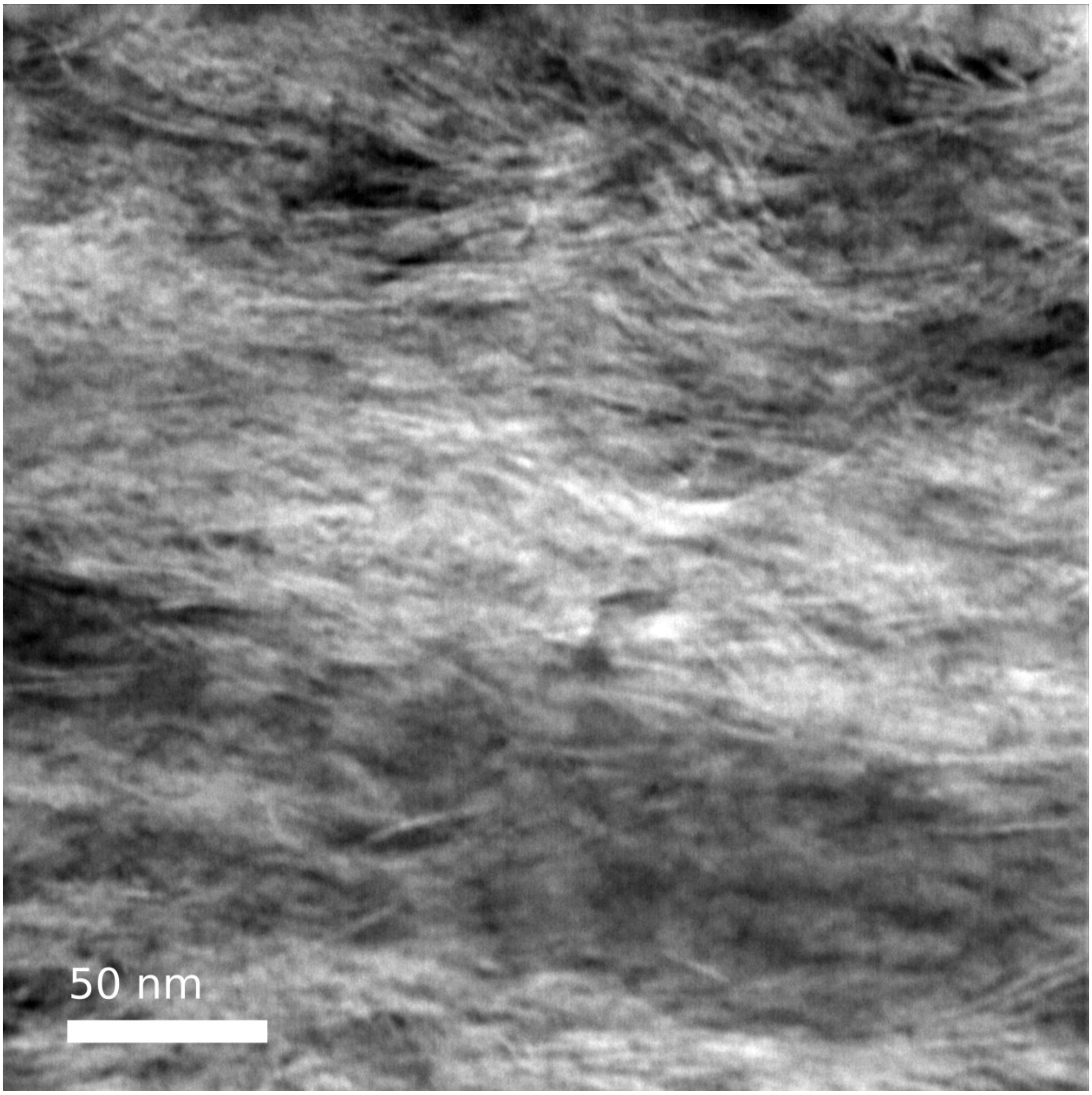


**Figure SI2 A representative STEM image of the nanostructure observed in the murine bone.**

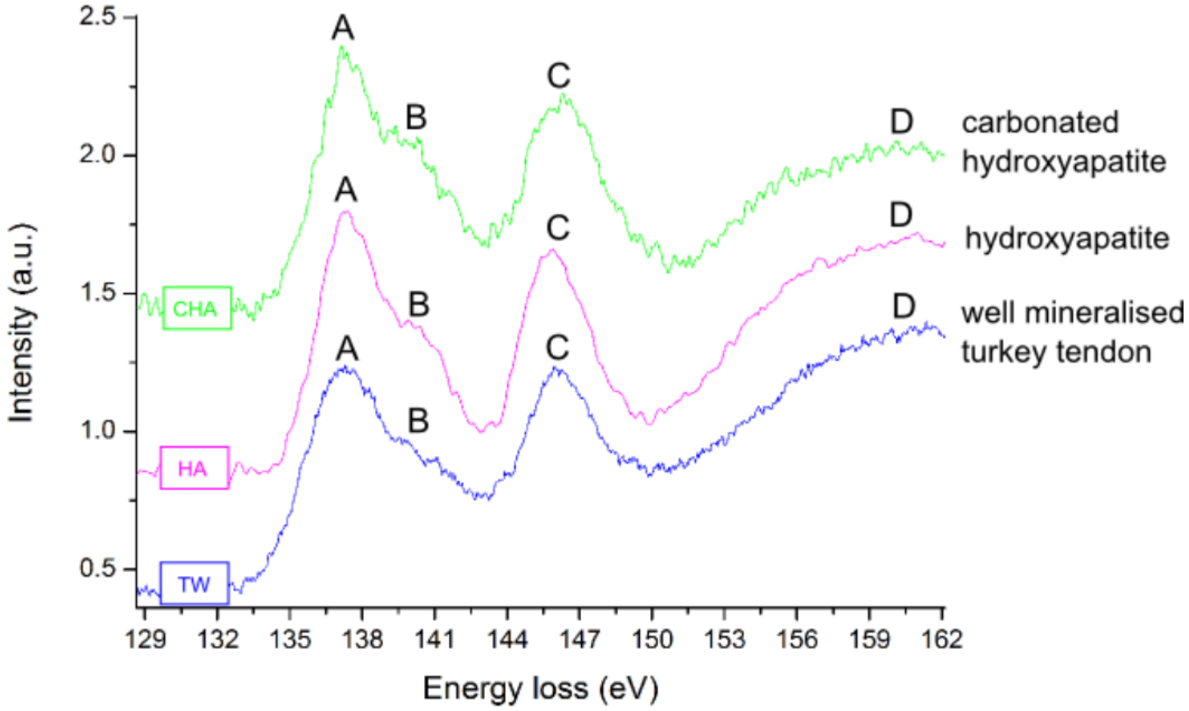


**Figure SI3 The phosphorus $L_{2,3}$ near edge structures of 14 week old well mineralised turkey tendon (TW) and the characteristic standards (HA – hydroxyapatite, CHA – carbonated HA). All turkey tendon patterns were consistent with patterns recorded for hydroxyapatite and carbonated hydroxyapatite. Turkey tendon regions in non-mineralised and in poorly mineralised regions did not exhibit a fully resolved phosphorous signal and have been omitted for clarity. A detailed review of the characteristic peaks A, B, C and D can be found in** [1,2]

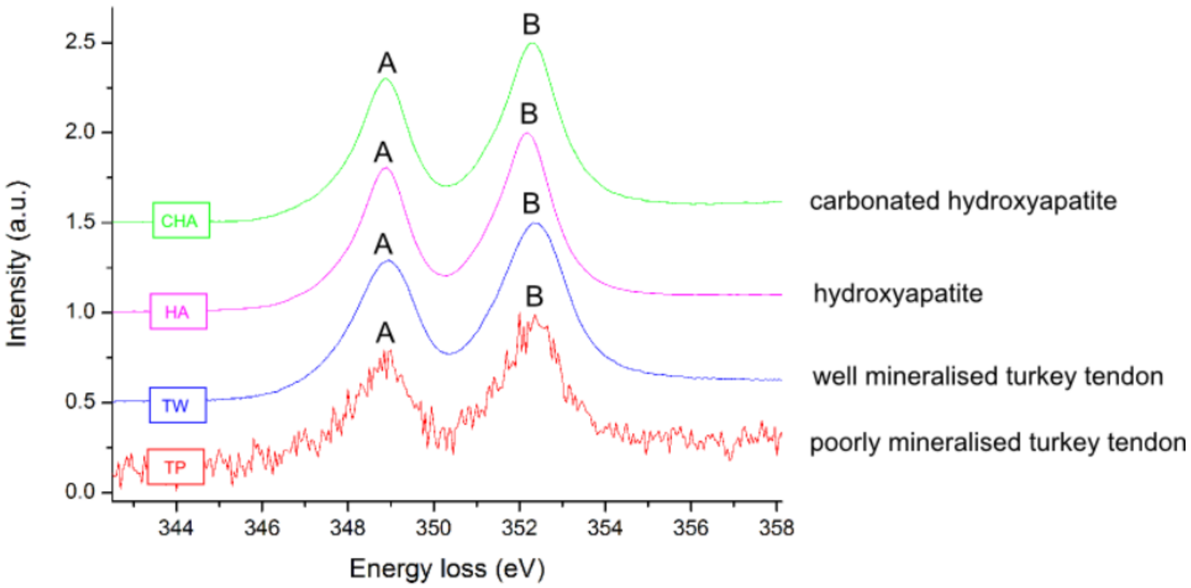


**Figure SI4 The calcium $L_{2,3}$ near edge structures of 14 week old mineralised turkey tendon (TP, TW) and hydroxyapatite (HA) and carbonated HA (CHA) standards. All calcium edge spectra exhibit two characteristic white line peaks: A at ~348 eV and B at ~351 eV.**

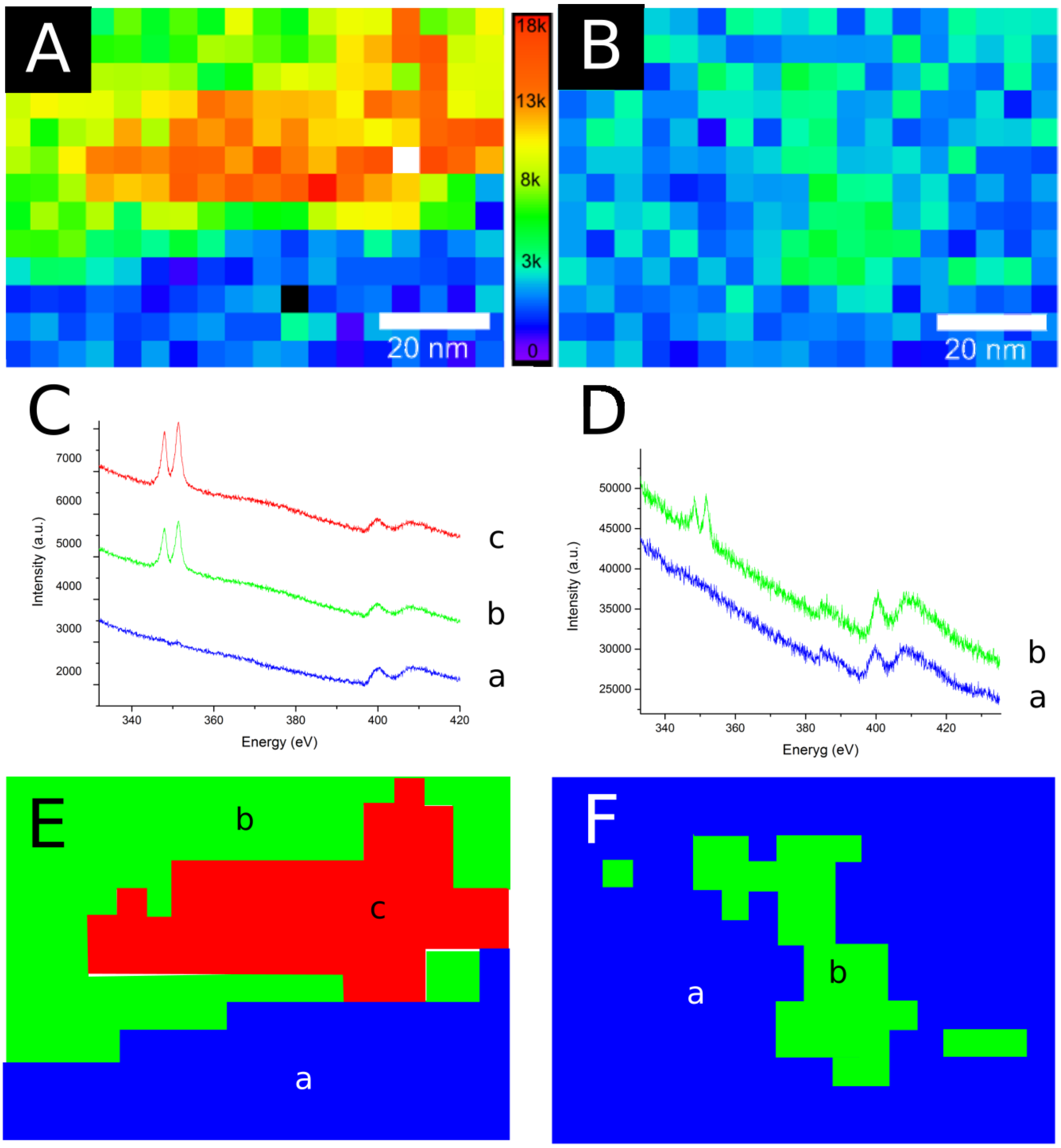


**Figure SI5 False-colour calcium intensity maps of (A) a large cluster and (B) a single granule as seen on Figure 2. Summed non-processed EELS spectra showing Ca-$L_{2,3}$ edge (C,D) taken from corresponding regions (E, F).**

**References**


1. Kłosowski, M. M. *et al.* Probing carbonate in bone forming minerals on the nanometre scale. *Acta Biomater.* **20,** 129–139 (2015).

2. Kłosowski, M. M. *et al.* Electron Microscopy Reveals Structural and Chemical Changes at the Nanometer Scale in the Osteogenesis Imperfecta Murine Pathology. *ACS Biomater. Sci. Eng.* (2016). doi:10.1021/acsbiomaterials.6b00300
